# Computational modelling as a tool in structural science

**DOI:** 10.1107/S2052252520011793

**Published:** 2020-08-29

**Authors:** C. Richard A. Catlow

**Affiliations:** a Department of Chemistry, University College London, 20 Gordon Street, London WC1H 0AJ, United Kingdom; b School of Chemistry, Cardiff University, Park Place, Cardiff CF10 3AT, United Kingdom

**Keywords:** structural science, computational modelling of crystal structures, structure prediction, editorial

## Abstract

This editorial gives a brief discussion of the current status and role of computational modelling as a technique in structural science.

Computational modelling is now integrated into almost all areas of science; and as well as being widely used for gaining insight and guidance in analysing and explaining experimental data, modelling has acquired an increasingly predictive capacity. This editorial will provide a brief review of its current status and role in structural science and will consider the likely future developments of the field. We focus first on our ability to model and predict crystal structures, after which we consider the challenges posed by disordered solids.

The use of computational methods in *modelling crystal structures* goes back many decades and in the 1970s and 1980s rapid progress was made with the use of methods based on interatomic potentials or force fields, coupled with energy minimization, to model accurately the crystal structures of a wide range of solids, both inorganic materials, including oxides, halides and silicates, and organic, molecular solids. These methods could be used to refine approximately known structures, as was also widely done in structural molecular biology. They could be enhanced by the use of molecular dynamical (MD) simulation methods and later by the use of density functional theory (DFT) based quantum mechanical methods, which as well as further refining crystallographic structures, generated models for the electronic structures of the materials. The article of Takada *et al.* (2018 ▸) provides an illustration of how MD methods can be used to gain insight into complex structures, in this case, those associated with tridymite, while many examples of the applications of DFT techniques will be found in recent issues of **IUCrJ**.

Structure modelling, based on known structures, which may be approximate, remains a useful tool. Far more challenging, however, is *structure prediction* based simply on the composition of the solid. Indeed, a celebrated challenge was issued over thirty years ago in a ‘News and Views’ article in *Nature* by John Maddox, who provocatively wrote: ‘*One of the continuing scandals in the physical sciences is that it remains impossible to predict the structure of even the simplest crystalline solids from a knowledge of their composition.’* The field has responded well to the Maddox challenge over recent years and successful structure prediction has now been achieved for many classes of material, as discussed in the reviews of Woodley & Catlow (2008 ▸), Price (2018 ▸), Oganov (2018 ▸) and Woodley *et al.* (2020 ▸). The general approach is to navigate the configurational space defined by the structural parameters, using a rapidly computable ‘cost function’ which may be a simple energy evaluation; regions of low cost function are identified and the resulting structures may then be refined using energy minimization coupled with a more accurate energy evaluation employing a force field or quantum mechanical technique. The navigation of configurational space can use a variety of techniques and algorithms, including simulated annealing, genetic algorithms, and topological and molecular packing approaches.

An elegant recent example of the success of structure prediction in inorganic materials is provided by the work of Collins *et al.* (2017 ▸), who used a Monte-Carlo-based simulated annealing algorithm to predict a new structure in the complex phase field of an Y–Sr–Ca–Ga-oxide; the material was subsequently synthesized and the predicted structure confirmed experimentally. Prediction methods have also been successfully applied to organic materials, especially porous organic solids as illustrated by the recent work of Pulido *et al.* (2017 ▸).

Structure prediction is also extensively used in nano-science as reviewed by Catlow *et al.* (2010 ▸) for nano-clusters of inorganic systems. Recent work of Lazauskas *et al.* (2018 ▸) provides a good illustration of the application to metallic nano-clusters with the structures for titanium clusters with 2 to 32 atoms predicted by combining an MC search procedure using a force field with DFT refinement. The resulting structures are shown in Fig. 1 ▸.

An important recent development is the growing use of machine learning techniques, as discussed by Woodley *et al.* (2020 ▸). More generally, the field continues to take advantage of both developments in technique and algorithms and the continuing expansion of computer power. Crystal structure prediction is still far from routine, but it is an increasingly important tool in structural science. As structure prediction becomes more widespread, it will be essential that the metadata accompanying the structure make it clear whether the structure is experimentally determined, predicted or some combination of the two.

Turning now to *disordered solids* there are many challenges to both experiment and computation as shown by several articles in **IUCrJ** over recent years. The first concerns the structures and energies of defects in crystalline solids. Modelling of *point defects* in solids was indeed one of the earlier successes of computational condensed matter science, where work in the 1970s and 1980s using force-field-based methods was able to achieve good agreement between calculated and experimental defect properties, especially in inorganic materials. The field was extended to include more complex defect structures, including point defect clusters and line and planar defects, and it has continued to develop rapidly. Contemporary work still makes some use of force-field methods, but is increasingly based on quantum mechanical methods using quantum mechanical/molecular mechanical (QM/MM) methods and periodic DFT techniques. Modelling is now an integral tool in the physics and chemistry of defective solids.

*Heavily disordered solids* including solid solutions and systems with high defect concentrations are attracting growing attention as again shown by articles over recent years in **IUCrJ**, and pose substantial challenges to theory and modelling. A common approach is to set up a supercell, aiming to model a disordered distribution within that cell. Realistic models may require very large cells and sampling of large numbers of configurations, although these requirements can be reduced by the use of symmetry as in the widely applied site occupancy disorder (SOD) approach developed by Grau-Crespo *et al.* (2007 ▸). Monte-Carlo techniques including those available in the knowledge led master code (KLMC) approach developed by Woodley and co-workers (see *e.g.* Lazauskas *et al.*, 2017 ▸) also assist in modelling complex disordered solids, but the field is one of the most difficult and demanding in structural science.

Computational methods have for many years been used to construct models of amorphous solids. The general approach is to mimic the way by which glasses have traditionally been made, that is to quench from the liquid state. The process is simulated by a molecular dynamics ‘melt quench’ cycle in which MD is used to simulate the melting of the crystalline form of the material, followed by rapid cooling in which the system freezes into the amorphous state. Due to limitations in the time sampled by even the most ambitious MD simulations, the method has the problem that the timescale of the MD quench is far shorter that of an experimental quench. A number of approaches have been developed to mitigate this problem and the MD modelling of disordered materials has enjoyed considerable success, although challenges remain. A detailed discussion of the field is given in the monograph of Massobrio *et al.* (2015 ▸); and there is no doubt that the structural science of amorphous solids will continue to gain valuable input and insight from modelling techniques.

The capabilities of modelling tools are advancing rapidly and the range and ambitions of applications will grow. **IUCrJ** will continue to welcome articles which develop and apply modelling techniques as a tool in structural science.

## Figures and Tables

**Figure 1:**
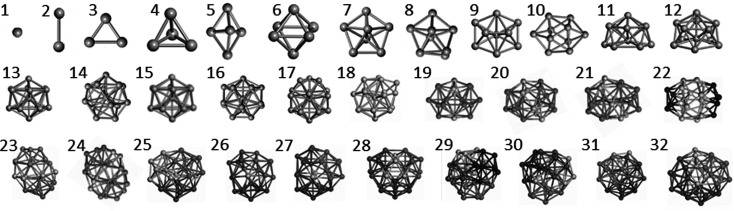
Predicted structures for titanium clusters with 2 to 32 atoms, after Lazauskas *et al.* (2018 ▸) (published by the PCCP Owner Societies).
